# Editorial: Solvation effects of organic reactions in ionic liquids, deep eutectic solvents, and conventional solvents

**DOI:** 10.3389/fchem.2023.1159357

**Published:** 2023-02-17

**Authors:** Ranjan Dey

**Affiliations:** Birla Institute of Technology and Science, Sancoale, India

**Keywords:** ionic liquids, conventional solvents, interactions, solvation effect, deep eutectic solvents

Solvent effects arise due to the effect of the solvent on the constituent component/s which is exhibited in the form of change in chemical reactivity and molecular dissociation or association. Its significance can be understood from the fact that the selection of the “correct” solvent may results in the formation of the desired products or complete failure in a given process. This selection is more pronounced in case of non-linear behavior being exhibited by the solution. Hence selecting the right solvent become critical in the case of Ionic Liquid (IL) mixtures which show large deviations from ideal behavior owing to their unique characteristics. These deviations affect the thermodynamic and transport properties of the liquid mixtures and solutions. These variations from ideal behavior can be understood through the study of the excess properties of the corresponding thermodynamic properties (Rama Rao et al., 2021) and through deviations in case of transport properties like viscosity, thermal conductivity, *etc.* Both ILs and Deep Eutectic Solvents (DES) are unconventional solvents and hence a thorough investigation will shed light on the various aspects of solvent effects in these systems (Qui et al., 2021). Recently Sunoj et al. (Das et al., 2022) have studied the molecular insights into the Solvation effects by employing Computational Chemistry tools in Organic reactions. Reichardt (2022) has given an overview on the physicochemical aspects of solvents involving molecular liquids, *viz.*, organic solvents, atomic liquids and ILs.

In this Research Topic, authors (Campodónico et al.) have carried out kinetic studies and reactive patterns in 1-chloro and 1-flouro- 2,4- dinitrobenzene with biothiols in aqueous medium and show the formation of electrophile/nucleophile adducts. From their findings authors draw the conclusion that the hydrogen bonding (HB) given by the reaction media and the reactivity patterns of the E+/Nu− pairs can be promoted by the ability of the solvent to accept or donate HB and their polarity.

Wang et al. have investigated the oxidation of 2-ethylhexanal (2-ETH) to 2-ethylhexanoic acid (2-ETA) and the solvent effects on the process. The effects have been studied though intermolecular forces and the interactions prevalent therein. The study also highlights the importance of polarity on reactivity and selectivity. The findings lead the authors to conclude that the very clear reactivity and product selectivity is obtained under specific solvent conditions. An increase in the solvent polarity shifts the intermolecular interaction from weak Van der Waals to stronger H-bonding.

Sanchez et al. have shed light on the Gutman’s donor and acceptor numbers based on binding enthalpy between solvent and probes. The investigation delves into the solvation effect on the reactivity of the ILs and DES. From their investigation and the results they conclude that a first-order theoretical model, based on the binding enthalpy between probes and solvents, is qualitatively reliable to embody solvent effects within a unified solvation effects model on chemical reactivity of ionic liquids.

Recently, Siami et al. have carried out studies on the effect on the cation functional group in SO_2_ acidic gas absorption by some amino acid ILs (AAIL). The authors carried out binding and Gibbs free energy studies of SO_2_ absorption and the findings showed that the most favourable green solvent was the AAIL functionalized by the COOH group as per thermodynamic data. DFT simulations employing Gaussian09 reversion A.01 was put to use for studying molecular structures, H-bond interactions and conformational properties.

These articles cover a broad spectrum of solvation effects of Organic reactions in ILs, DES and conventional Solvents and will benefit the readers in helping them to choose the “right” solvent for their specific processes and applications.
